# Environmental and biological drivers of movement activity in *Callimico goeldii* under zoo conditions

**DOI:** 10.1038/s41598-025-18540-7

**Published:** 2025-09-26

**Authors:** Zenon Nieckarz, Jacek Nowicki, Malwina Lasko, Krzysztof Pawlak

**Affiliations:** 1 Department of Experimental Computer Physics, Institute of Physics, Jagiellonian University, ul. prof. Stanisława Łojasiewicza 11, 30-348, Kraków, Poland; 2 Department of Genetics, Animal Breeding and Ethology, University of Agriculture in Cracow, Aleja Adama Mickiewicza 24/28, 30-059, Kraków, Poland; 3 Department of Zoology and Animal Welfare, University of Agriculture in Cracow, Aleja Adama Mickiewicza 24/28, 30-059 Kraków, Poland

**Keywords:** Behavioural methods, Bioinformatics, Imaging, Software

## Abstract

Animal welfare is a crucial aspect of zoological research, with behavioral assessment serving as a key indicator of well-being. This study aimed to evaluate the effects of photoperiod duration, daylight savings shift from winter to summer time, atmospheric pressure, and the occurrence of parturition on the motor activity of *Callimico goeldii* under zoo conditions. Animal activity analysis was performed via an automatic image analysis method in a family group of nine individuals over two months. Earlier sunrise was associated with an earlier onset of activity, whereas the lengthening of daylight in the afternoon did not affect the extension of motor activity at the end of the day. A decrease in the motor activity of the group was observed after parturition. The demonstrated upward trend in motor activity following the shift from winter to summer time likely resulted from stress due to changes in the timing of *Callimico goeldii* handling. The results also showed that daily fluctuations in atmospheric pressure constitute a strong stimulant for Callimico goeldii and may cause changes in the functioning of these primates. In conclusion, it can be stated that the environmental and biological factors considered in this analysis can significantly influence the activity patterns of this species and should be taken into account when assessing their welfare under zoo conditions.

## Introduction

According to the classical definition, animal welfare is the state of an individual, determined by its capacity to adapt to the environment, encompassing a broad spectrum of coping strategies^1^. The most well-known current model of animal welfare is the Five Freedoms Model^2^, nonetheless, positive welfare has recently become increasingly popular. In contrast to approaches that focus solely on reducing suffering, positive welfare broadens the scope of animal well-being. The concept emphasizes the opportunity for animals to express species-typical behaviors driven by intrinsic motivation, ensuring a good quality of life, fostering positive emotions, and enhancing overall happiness^3^. Animal behavior is a crucial indicator in assessing the level of animal welfare. Ethograms commonly include mobility as a fundamental behavioral element, operationalized as an animal’s activity level within a defined temporal frame. As a key attribute of biological systems, movement plays a pivotal role in structuring and modulating their dynamics, ranging from individual- to ecosystem-level behaviors^4^. The method proposed by Nieckarz et al.^5^ represents one approach for assessing animal mobility. Specifically, this methodology enables the registration and analysis of behavioral changes in rapidly moving animals.

Weather factors may influence animal behavior^6,7^. One element impacting animal behavior is light. Sunlight on Earth and the associated day and night cycles affect the periodicity of physiological processes in living organisms, influencing reproduction, development, and metabolism^8,9^. As shown by Shuboni et al.^10,11^, sunlight also affects mammals’ circadian rhythm activity. Atmospheric pressure can also affect animal functioning. Atmospheric pressure is classified as a mechanical biometeorological stimulus, and the stimulatory effects are caused primarily by fluctuations in atmospheric pressure over a short period^12^ and above-average high or low pressure values^13^.

Large fluctuations in atmospheric pressure may affect not only the physiology of organisms^14^, but also behavioral responses in animals^15,16^.

In addition to weather factors, animal behavior can be influenced by parturition and the arrival of a new member to the herd. *Callimico goeldii* is a notably prevalent species in many zoological gardens. This South American primate inhabits the tropical rainforests of the upper Amazon region^17^. It typically lives in small groups, averaging approximately 4–5 adults, with group sizes ranging from a minimum of 2 to a maximum of 12 individuals^18^.

Due to the large variety of species inhabiting relatively small spaces and the limited knowledge of their specific needs, assessing the welfare of animals in zoos is particularly difficult. Hence, science-based tools to assess and monitor animals are needed to protect and promote their welfare in zoos^19^.

Therefore, this study aimed to evaluate the effects of photoperiod duration, the shift in daylight from winter to summer, atmospheric pressure, and the occurrence of parturition on motor activity in *Callimico goeldii* under zoo conditions.

## Materials and methods

### Ethical statement

Our study (permit no. 29/2015) involved noninvasive, camera-based monitoring of the animals, ensuring that no new factors were introduced into their environment. According to directive 2010/63/EU of The European Parliament and the Council of the European Union (22 September 2010) on the protection of animals used for scientific purposes, such activities do not require approval from an ethics board.

The Animal Welfare Committee at the Faculty of Animal Science, University of Agriculture in Krakow, confirmed that the research described in this publication did not require approval from the local ethics committee for animal experimentation.

Silesian Zoological Garden granted permission to conduct the research described in this article.

### Research subjects

We collected behavioral data for one particular group of animals - a family of *Callimico goeldii* monkeys. The family comprised nine animals: five females and four males. Since March 14, as a result of birth, the number of animals has increased to 10 individuals. The species Callimico goeldii was selected as a model organism not only due to its availability in zoological gardens, but also because of its specific ecological characteristics. It is a mammal native to the equatorial regions of South America, where the duration of day and night remains nearly constant throughout the year. Keeping these animals for several generations in conditions where the photoperiod changes seasonally, enables the investigation of how animals originally adapted to a stable light–dark cycle, respond to its variability.

### Housing

The indoor enclosure for *Callimico goeldii* was situated within the monkey house pavilion at the Silesian Zoological Garden in Chorzów, Poland. The dimensions of the enclosure measured 4 m × 2.85 m. Glazed windows built into the walls allowed sunlight to enter the interior of the room where the monkeys were housed.The walls were constructed using decorative elements designed to mimic rocks to imitate the landscape and climatic conditions of the species’ natural habitat. .The enclosure was separated from the visitor corridor by a glass partition. The temperature and humidity in the enclosure were maintained at 24 °C and 75%, respectively. For temperature regulation, four heat-emitting radiators were installed and enclosed in a metal mesh for safety. Humidity was monitored daily and increased as needed through manual spraying of the substrate on the floor. Pine bark was used as a substrate to retain moisture. The enclosure was equipped with permanently installed wooden shelves that facilitate locomotion and jumping for the animals, along with various thin and thick branches serving as structural and decorative elements. In addition, small conifers and deciduous trees harvested from the garden were placed in the enclosure seasonally. Efforts were made to ensure that the room contained many living plants, which also helped maintain an appropriate humidity level. Artificial lighting was always (according to both winter and summer time ) turned on at 7:00 am and turned off at 5:00 pm.

### Husbandry and feeding

Daily husbandry routines were consistent throughout the study period. The animals were fed twice a day at fixed times – in the morning and early afternoon. Their diet included fruits (e.g., apples, bananas, grapes), vegetables (e.g., carrots, courgettes), insects (e.g., mealworms, crickets), cooked eggs, and edible mushrooms (e.g., oyster mushrooms). The diet was periodically supplemented with calcium and vitamin preparations.

Environmental enrichment was provided regularly. It included both structural elements, such as ropes, branches, platforms, and feeding-based enrichment, such as hiding insects or food items in natural materials. These measures aimed to promote species-typical behaviors and enhance animal welfare.

The animals were cared for by trained zookeepers as part of routine husbandry. No direct physical handling was involved outside of basic care procedures, and no handling took place during the observation period.

### Period of analysis

The study was conducted from 01.03.2016 to 29.04.2016. The observations on 14.03.2016 and 15.03.2016 were stopped at the zoo’s request. The study distinguished the time before parturition (from 04.03.2016 to 13.03.2016) and after parturition (from 17.03.2016 to 29.04.2016) and the period before (from 01.03.2016 to 26.03.2016) and after (from 27.03.2016 to 29.04.2016) the transition from winter to summer time. Throughout the manuscript, the terms ‘daylight saving time (DST)’ and ‘standard time’ are used instead of ‘summer time’ and ‘winter time’, to refer to time changes associated with official clock adjustments, not to seasonal periods. The “prenatal period” and “postnatal period” refer to the time directly before and after the parturition, the date of which was determined based on direct observations.

### Measuring equipment and gathered data

This study was based on observations of the *Callimico goeldii* family. Video recordings were obtained via a BCS AT V554OSDIR48 digital camera operating in the PAL system with a 1/3” SONY CCD sensor. The image resolution was 704 × 576 pixels (width × height), and the frame-per-second rate was 14 Hz. Detailed descriptions of the equipment can be found in Nieckarz et al.^5^. The camera was placed in the upper region of the room at a height of 3.2 m and was oriented toward the shelves where the monkeys most frequently congregated and interacted. Additionally, the camera was equipped with supplementary infrared lighting to enable the observation of the animals at night.

Animal activity analysis was performed via an automatic image analysis method^5^, with the A1 index as the final parameter. The A1 index is a quantitative measure of the motor activity of the observed animal group, calculated automatically from video recordings analysed frame by frame. The algorithm compares each successive frame with a background model generated from the preceding 28 frames (approximately 2 s). For each pixel, the Mahalanobis distance^20^ in grayscale is calculated to detect changes. Grayscale image analysis allows for the standardization of material recorded in daylight (color images) and at night using an infrared illuminator (grayscale images). If the calculated value for a given pixel exceeds the predetermined threshold, the pixel is classified as “active” (movement) and assigned a white value, while all other pixels remain black (no movement).

This process produces a binary motion mask for each frame, from which the proportion of white pixels in the entire image is calculated, yielding the value of the A index for that frame. The A1 index is then computed as the fraction of time (e.g., within a minute, hour, or day) during which the A index exceeds the experimentally determined threshold of 0.01^5^, corresponding to periods of noticeable group motor activity. The threshold of 0.01 is used to suppress noise and random fluctuations in the image. In summary, the A1 index indicates the proportion of the observation period during which group activity was detected.

The described method, based on the A1 index, measures the activity of the entire animal group and, unlike approaches such as overall dynamic body acceleration (ODBA), does not require any intervention in the animals or their environment. Because the A1 index method relies on the analysis of changes in video recordings, it enables the monitoring of group activity while also capturing limb and head movements, as well as other subtle behaviors that ODBA, based on accelerometers mounted on the animal’s body, would not detect. In practice, the A1 method is particularly useful for group studies conducted in enclosed and continuously monitored enclosures, whereas ODBA is more suitable for precise monitoring of individual animal activity, including in field conditions where video recording is difficult or impossible.

The analysis included the activity of the entire group, without distinguishing between individual animals, because the system records the movement of the group as a whole.

Atmospheric pressure measurement data from the Katowice-Muchowiec synoptic station located 5 km from the zoo were obtained from the following website: https://danepubliczne.imgw.pl. The average daily pressure values (p) and pressure changes from day to day were calculated using these data (Δp_i_ represents the difference between the pressure on the i-th day (p_i_) and the pressure on the previous day (p_i_-1)).

The sunrise and sunset times and the length of daylight hours were determined via a dedicated calculator available at https://gml.noaa.gov/grad/solcalc/sunrise.html.

### Statistical analysis

OriginPro 2022b (9.9.5.167, Academic) was used for the statistical analyses and to graphically represent the data. The graphs present the mean values and respective standard deviations. Linear regression was employed to evaluate the trend of changes. A regression coefficient significance test (Student’s *t* test) was performed to determine if the slope of the linear fit significantly deviated from zero. Pearson’s correlation coefficient was applied to assess the strength and direction of the relationships between variables. Differences between the mean values of the variables were examined via the *t* test, whereas the F test was used to compare the variances.

### Results and discussion

**Fig. 1:**
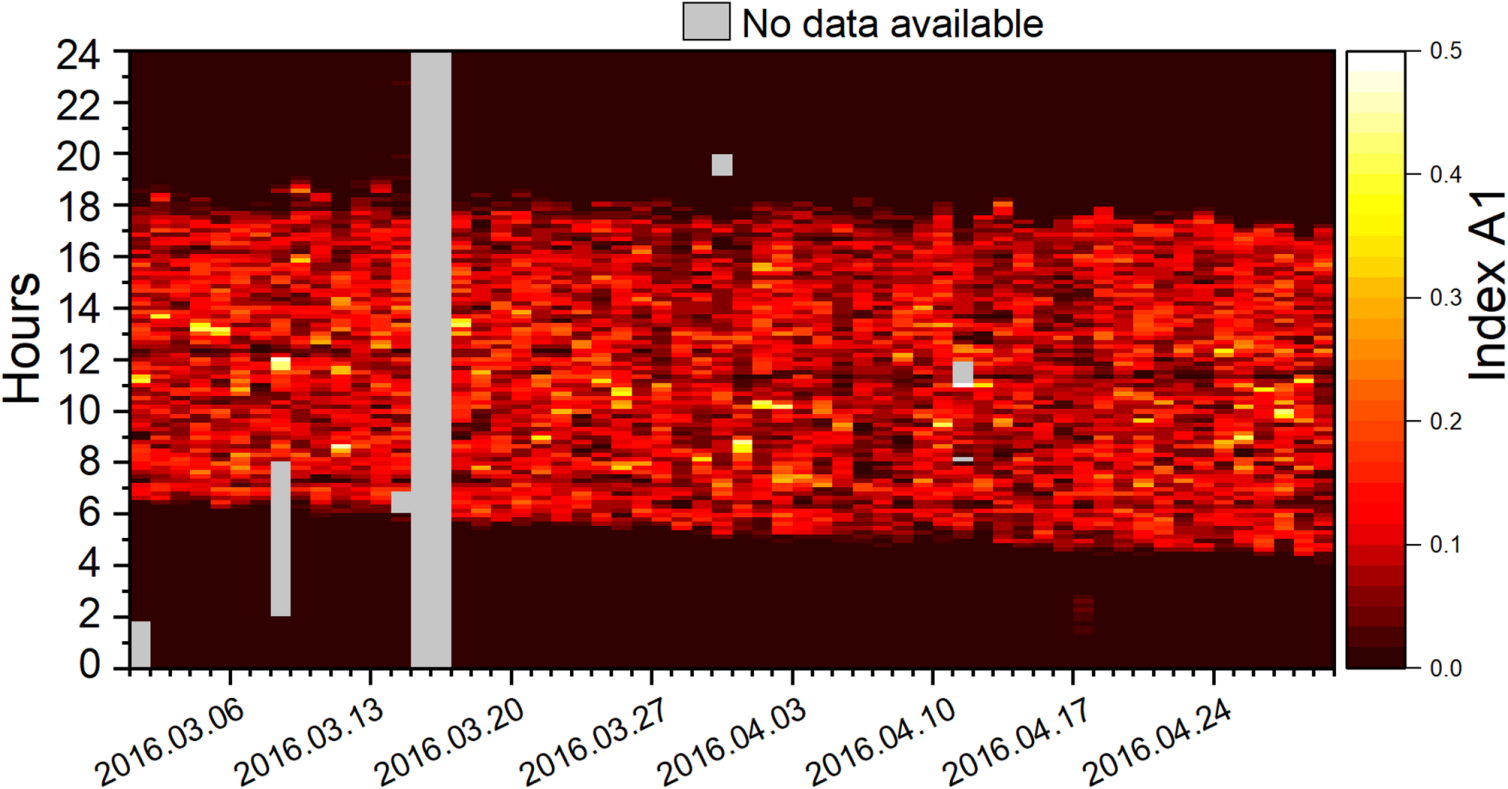
Distribution of the A1 activity index as a function of hours (vertical axis, 10-minute resolution) and days (horizontal axis, 1-day resolution). The data presented were collected in winter time.

Life on Earth has evolved in the presence of diurnal cycles for millions of years, leading to the development of biological systems in organisms whose health and well-being are fundamentally dependent on physiology and behaviors adapted to this rhythmic environment. While locomotor activity and sleep display the most evident circadian patterns, numerous other physiological and behavioral processes crucial for survival, including hormone regulation, body temperature in endotherms, metabolism, and immune function, are also governed by rhythms. The coordination of these rhythms in organisms depends on exposure to light and darkness^21^. Figure 1 presents the changes in the A1 index over successive days of observation.

 An earlier onset of activity in *Callimico goeldii* is observed with sunrise. On the first day of measurement, 01 March 2016 (sunrise 6:25 AM winter time), activity started at approximately 6:30 AM, whereas on the last day of the measurement period, 29 April 2016 (sunrise 5:25 AM summer time, or 4:25 AM according to the winter time marked in Fig. 1), activity started at approximately 4:30 AM winter time. This change in activity is most likely associated with the activation of suprachiasmatic nucleus (SCN) structures located in the mammalian brain by sunlight. SCN structures are directly connected to the retina and activated by incident light from the rising sun, causing the onset of *Callimico goeldii* activity^22^.

 Although the animals were housed indoors under stable artificial lighting conditions, natural daylight did reach the enclosure through glazed windows. This early morning light likely acted as an indirect zeitgeber. Alongside other cues, such as the appearance of caretakers or feeding times, it may have contributed to the observed shift in activity onset. While the influence of natural sunlight cannot be isolated with certainty, it should be considered among the environmental factors affecting circadian behavior.

 However, no changes in activity associated with the lengthening of daylight in the afternoon were observed, taking into account that at the beginning of the measurements (1 March) sunrise was at 06:25, sunset at 17:26 (winter time) and at the end (29 April) sunrise was at 05:21 and sunset at 20:01 (summer time).This is most likely because, in the afternoon, there were no stimuli that could excite the monkeys (e.g., cleaning, feeding, treatments, and visits from caregivers). Additionally, for *Callimico goeldii* under natural conditions, the hours between 5 PM and 6 PM are a period of rest and preparation for sleep^18^. The increase in activity (A1) recorded at night on 17 April (between 1:10 AM and 3:00 AM) is related to the movement of rodents in the observed room, which aroused the monkeys.

**Fig. 2:**
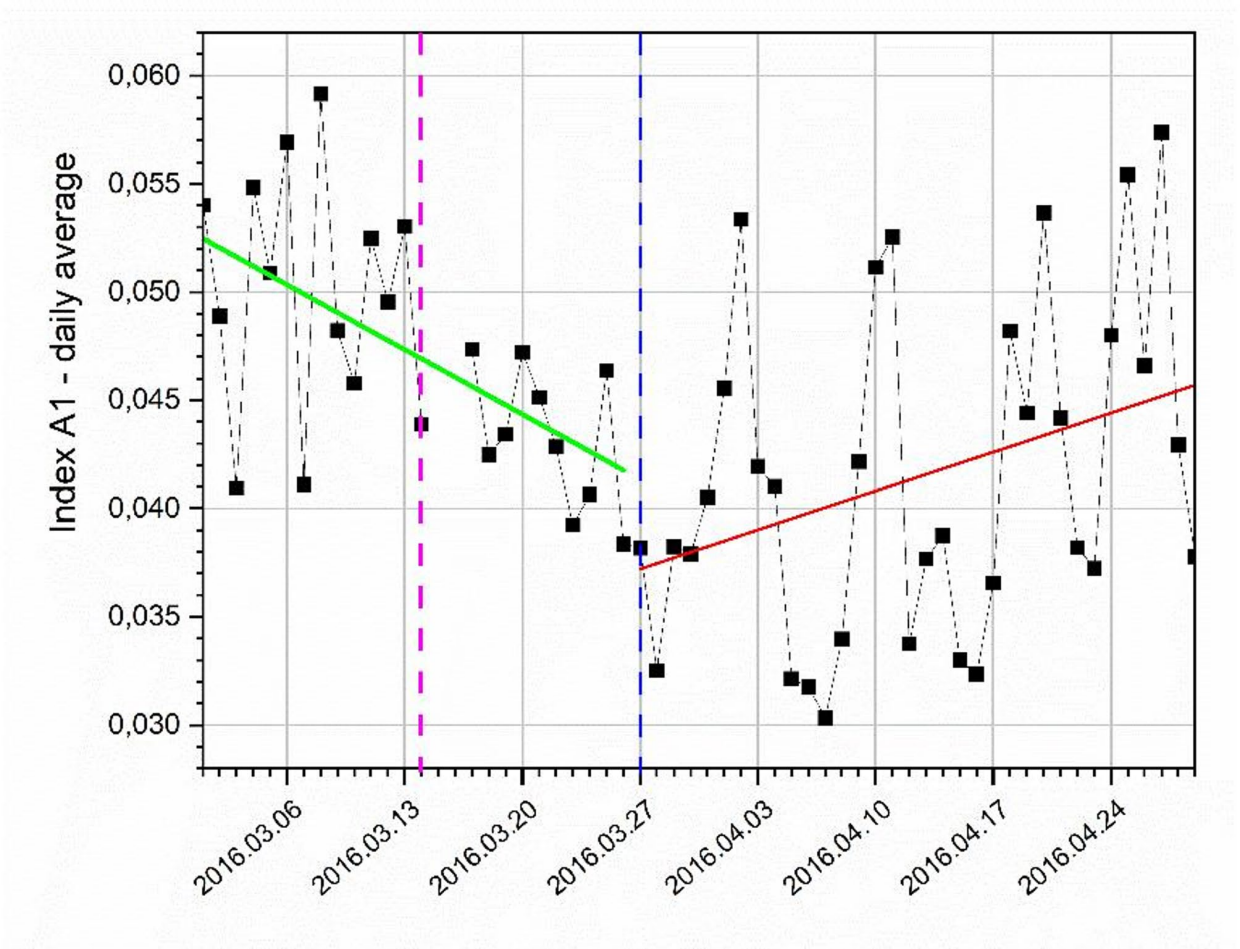
Course of the average daily activity index (A1) throughout the study. The vertical dashed purple line indicates the day of parturition, and the dashed blue line indicates the day of the time change. The green line represents a linear fit to the data from the period when the standard time (winter time) was in effect (slope = − 4.28 (± 1.24) × 10⁻⁴), and the red line represents a linear fit when the daylight saving time (summer time) was in effect (slope = 2.57 (± 1.26) × 10⁻⁴).

 When assessing the average daily activity of the monkeys during the entire study period (Fig. 2), the linear fit for the winter period data revealed a negative slope (the green line in Fig. 2), whereas the summer period data demonstrated a positive slope (the red line in Fig. 2) at the 0.05 significance level. This finding indicates a decrease in activity in the first period and an increase in the second period. The difference between the mean A1 values for the winter and summer periods was significant (*t* test, *p***-** value = 0.003), whereas the variances were not significantly different (F test, *p- value* = 0.18).

From the day of the shift from winter to summer time, the staff appeared one hour earlier in the building where the *Callimico goeldii* monkeys were kept and food was served. Thus, the stable schedule of monkey care that had been maintained for many months was disrupted. For the observed individuals accustomed to a constant daily rhythm, such a modification may have been a stress stimulus, which is reflected in the change in the animals’ activity (Fig. 2). There is a lack of research on the effects of the transition from winter to summer time on animals, and studies involving humans remain limited. As Hadlow et al.^23^ demonstrated, changes in time, especially from winter to summer, cause disruptions in the functioning of the body, which may persist for several months. Changes in behavior in both adults and adolescents were also observed by Kamstra et al.^24^ and Medina et al.^25^. Manfredini et al.^26^ described several stress factors that appear when the time changes, affecting the body and causing numerous disorders in the circulatory system and changes in human behavior. Stults-Kolehmainen & Sinha^27^ summarized 168 studies by various authors on the effects of stress on human activity, clearly revealing that stressful situations significantly affect locomotor activity. The reports cited above are consistent with the hypothesis that the increase in physical activity in animals observed after the change from winter to summer might be related to stress resulting from daily routine changes.

 In addition to the shift from winter to summer time, parturition could affect *Callimico goeldii* monkey activity.

**Fig. 3:**
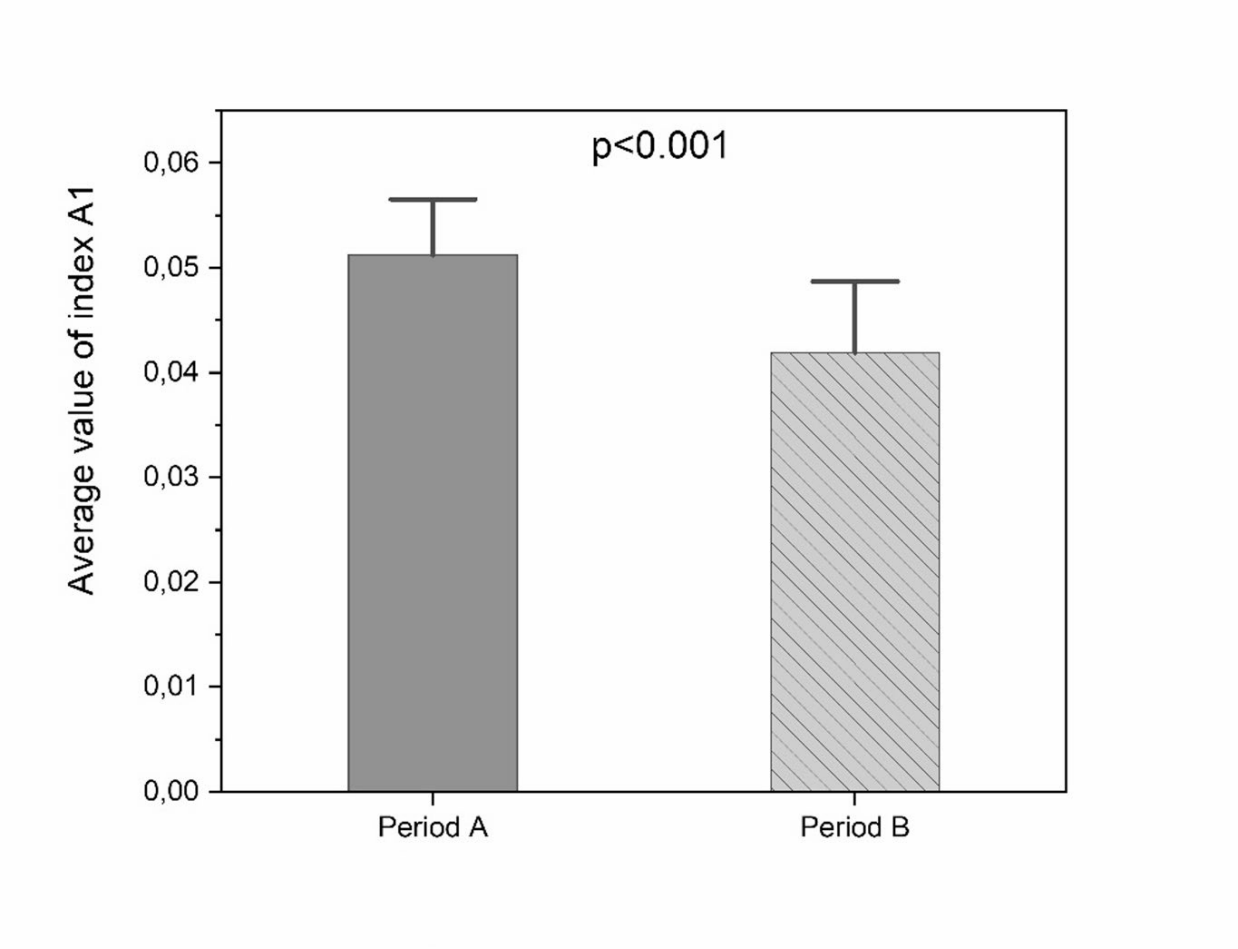
Average values of the A1 activity index with corresponding SDs in the periods before (Period A) and after (Period B) parturition.

As shown in Fig. 3, there was a significant difference in activity (A1) between the prenatal and postnatal periods, where the mean values of the indices were 0.0512 (SD = ± 0.0053) and 0.0419 (SD = ±0.0068), respectively (*p- value* < 0.001).

 Taking into account both Figs. 2 and 3, it can be observed that the birth of the infant led to a noticeable change in group activity. The increase in activity that followed the transition from standard time to daylight saving time was interrupted by the parturition event, initiating a declining trend in group activity (green regression line in Fig. 2).

 The changes in monkey behavior after the appearance of a newborn, as shown in Fig. 3, are also supported by limited previously published studies. Mothers devote a significant quantity of time to their offspring in the initial period of life, remaining as close to them as possible. However, in the later stages, mothers encourage their young to become increasingly independent^28,29^.

 The activity of other group members also changes in response to a new birth. In some species, such as colobus monkeys, females allow their newborn infants to be held and carried for long periods by other group members (infant care)^30,31^. In other animals, such as baboons and macaques, mothers are much more restrictive in allowing access to their young offspring^30,32^, despite persistent attempts by group members to interact with the infant^33^.

**Fig. 4:**
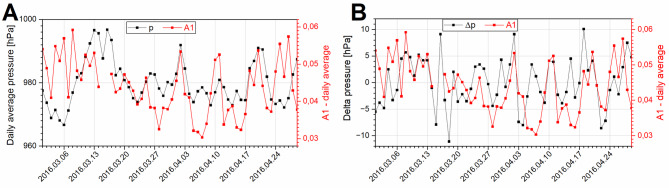
Course of average daily values of the A1 index and atmospheric pressure p (panel **A**) and the daily values of the A1 index and changes in pressure Δp (panel **B**).

The analysis of the relationship between the physical activity of the studied animals and atmospheric pressure revealed no correlation between the A1 index and the pressure value (*R* = 0.13, *p- value* = 0.34). However, a correlation was found (*R* = 0.29, *p- value* = 0.02) between the A1 index and the change in pressure Δp, where Δp_i_ is the difference between the pressure on the i-th day (p_i_) and the pressure on the previous day (p_i−1_).

 Changes in external environmental conditions necessitate adjustments in organism function to restore homeostasis. Notably, atmospheric pressure has the same effect on individuals inside and outside buildings^34^. The change in atmospheric pressure from day to day is a strong, adverse mechanical stimulus organisms^35^. A sudden change in atmospheric pressure causes serious disturbances in the functioning of the nervous system and the balance of internal pressure. Such disruptions may lead to changes in behavior, manifesting as excessive arousal^36^, which was visible during our observations of *Callimico goeldii* (Fig. 4, panel B).

### Limitations

One limitation of this study is the lack of quantitative data on zoo visitor attendance during the observation period. Although the animals were housed in indoor enclosures separated from the public area by glass panels, and the birth of the infant was not promoted by the zoo, it cannot be ruled out that human presence near the enclosures—particularly the activity of zookeepers and technical staff—may have influenced the animals’ behavior.

 However, the issue of human presence and its effect on animal behavior, including both staff and visitors, is broad and complex. It requires a separate, in-depth analysis based on a large dataset. For this reason, this topic has been excluded from the current study and is the subject of a separate manuscript currently in preparation.

## Conclusion

The results indicate that the factors analyzed in this study significantly influenced the behavior of the observed individuals. It was observed that an earlier sunrise led to an earlier onset of activity, whereas the extension of daylight in the afternoon did not prolong motor activity at the end of the day.

The increase in activity following the transition to daylight saving time likely resulted from stress induced by a complex set of environmental and organizational factors. These factors changed alongside the time shift and collectively altered the daily rhythm and living conditions of Callimico goeldii.

Parturition caused a marked decrease in the group’s motor activity. This effect may have offset the increase in activity observed after the transition to daylight saving time, indicating a complex interaction between biological and environmental factors affecting Callimico goeldii behavior.

It should be noted, however, that the postnatal period partially overlapped with the time following this change, complicating the clear interpretation of these results.

Additionally, the findings demonstrated that daily fluctuations in atmospheric pressure constitute a significant stimulus for Callimico goeldii and may lead to changes in their functioning.

Consequently, it can be concluded that both environmental and biological factors substantially affect the activity patterns of this species and should be taken into account when assessing their welfare in zoological settings.

Further research is needed to elucidate the individual effects of each analyzed factor on Callimico goeldii activity patterns and to identify the physiological mechanisms underlying behavioral responses to atmospheric pressure changes.

## Data Availability

All data generated or analyzed during this study are described in this published article. All materials are housed at the Department of Zoology and Animal Welfare, University of Agriculture in Krakow, Krakow, Poland. The data supervisor is Professor Krzysztof Pawlak. The datasets used and/or analyzed during the current study are available from the corresponding author(s) upon reasonable request.
